# Supplementary material for: Antimicrobial, Antibiofilm, and Antioxidant Properties of Essential Oil of Foeniculum vulgare Mill. Leaves
Source: Plants (Basel). 2022 Dec 17;11(24):3573. doi: 10.3390/plants11243573 (PMC9783700; doi:10.3390/plants11243573)
Supplement: Supplementary file 1 [file plants-11-03573-s001.zip › plants-2088787-supplementary.pdf]

Table S1. Chemical composition of the EOs of leaves of *F. vulgare* subsp. *vulgare* var. *vulgare* from Sicily.

No.	Component	LRI[a]	LRI[b]	Area (%)
**1**	*α*-Pinene[c]	938	1025	33.75
**2**	Sabinene[c]	974	1123	3.63
**3**	*β*-Pinene[c]	976	1102	5.13
**4**	Myrcene[c]	989	1154	5.25
**5**	*α*-Phellandrene	1001	1158	2.05
**6**	*δ*-3-Carene	1010	1140	6.12
**7**	*p*-Cymene	1051	1243	9.45
**8**	Fenchone	1081	1391	1.85
**9**	(*E*,*Z*)-2,6-Dimethyl-2,4,6-octatriene	1130	1382	0.26
**10**	*exo*-Fenchol	1135	1598	0.14
**11**	*trans*-Sabinol	1144	1720	0.10
**12**	4-Terpineol[c]	1176	1601	0.07
**13**	Estragole	1187	1677	25.06
**14**	Fenchyl acetate	1207	1468	0.13
**15**	(*E*)-Anethole[c]	1300	1833	5.30
	**Monoterpene hydrocarbons**			**65.64**
	**Oxygenated monoterpenes**			**2.29**
	**Phenylpropanoids**			**30.36**
	**Total**			**99.83**

[a] Linear retention index on a HP-5MS column; [b] Linear retention index on a DB5 column; [c] Co-elution with authentic sample.
